# Diseases of the Upper Respiratory Tract

**Published:** 1895-03

**Authors:** 


*Diseases of the Upper Respiratory Tract.* By P. Watson Williams, M.D. Pp. xvi., 282. Bristol: John Wright & Co. [1894.]

This manual, written by a physician, deals especially with the medical aspect of rhinology and laryngology, while at the same time a good outline of the surgical treatment is added, so as to make it a complete work of reference for the student or practitioner. In such a work we naturally find prominence given to such subjects as "throat affections of infectious fevers, gout, and rheumatism"; "chronic infective diseases," such as syphilis, tuberculosis, and leprosy; and "neuroses of the larynx": accordingly there are useful chapters on each of these topics, while shorter descriptions are given of the more purely manipulative and operative procedures such as tracheotomy, tonsillotomy, and thyrotomy. We notice, however, a very good and practical description of intubation, of which the author speaks with discriminative approval, especially recommending it for laryngeal obstruction the result of acute laryngitis in children in all cases "where medical aid can be summoned within half-an-hour should the tube be coughed up." Comparatively little space is devoted to the subject of diphtheria, inasmuch as it "is treated at length in every text-book of general medicine." We think, however, that any work on throat diseases ought not to omit a description of the Klebs-Loeffler bacillus and a figure of its microscopical appearance. The author very properly remarks that "there is no longer any doubt as to the occurrence of membranous croup distinct from diphtheria," but he hardly insists enough upon the fact that the only positive distinction between the two is the bacteriological one.

The book is illustrated with five coloured plates as well as numerous woodcuts, all drawn by the author himself and most of them from nature, while the frontispiece shews a beautiful dissection in the museum of Bristol University College illustrating the anatomical relations of the interior of the nose, naso-pharynx, pharynx, and larynx. Most of the illustrations are distinctly good, and greatly add to the value of the book. It is written in a pleasant easy style, and is particularly well adapted for students commencing the study of throat diseases.

